# Concentrating white spot syndrome virus by alum for field detection using a monoclonal antibody based flow-through assay

**DOI:** 10.14440/jbm.2016.105

**Published:** 2016-05-25

**Authors:** Amrita Rani, Sathish R. Poojary, Naveen Kumar B. Thammegouda, Abhiman P. Ballyaya, Prakash Patil, Ramesh K. Srinivasayya, Shankar M. Kalkuli, Santhosh K. Shivakumaraswamy

**Affiliations:** College of Fisheries, Karnataka Veterinary Animal and Fisheries Sciences University, Mangaluru – 575002, Karnataka, India

**Keywords:** alum, field detection, flocculation, flow-through assay, WSSV

## Abstract

A simple and easy method of concentrating white spot syndrome virus by employing aluminium sulphate, alum as a flocculant was developed and evaluated for field detection. The concentrated virus was detected by a monoclonal antibody based flow-through assay, RapiDot and compared its performance with polymerase chain reaction. The semi-purified virus that was flocculated by 15 and 30 ppm alum in a 50 ml cylinder can be detected successfully by both RapiDot and I step PCR. In addition, alum could also flocculate the virus that is detectable by II step PCR and the concentration of virus was similar to the one observed in water from an infected pond. Furthermore, experimental infection studies validated the successful concentration of virus by alum flocculation followed by rapid detection of virus using monoclonal antibody based RapiDot. Overall, the results obtained in this study indicate that the white spot syndrome virus in water could be easily flocculated by alum for sensitive field detection by monoclonal antibody based RapiDot.

## INTRODUCTION

The intensification of shrimp culture and its expansion has been largely increased over the decades to meet the global demand and has simultaneously resulted in the increase in number of viral pathogens to the OIE list for penaeid shrimps. Among the OIE list of shrimp viruses, white spot disease (WSD) caused by white spot syndrome virus (WSSV) has the largest impact and continued to be an obstacle to the sustainable shrimp farming [1]. WSSV is an envelope, bacilliform, and ovoid virus measuring 80–120 nm in diameter and 250–380 nm in length with a rod–shaped nucleocapsid containing dsDNA with an estimated size of 290–305 kb [2-5]. The WSSV has been known to transmit horizontally through water and feeding on infected shrimps [6, 7], and vertically through brooders to offspring [7, 8]. As such there are no treatments to control this disease and hence, the only alternative available is to avoid the entry of WSSV into shrimp culture practices, might be through stringent biosecurity measures [9]. Water is one of the major components of commercial shrimp farming and probably is the main route of entry for WSSV into an aquaculture facility [10-12]. The routine practices of shrimp farming including the wastewater discharge from ponds into the adjacent lagoons and estuaries, and heavy water exchange during WSD outbreak increases the risk of WSSV transmission to the neighboring shrimp farms through water. Therefore, the best possible way to avoid the entry is by detecting WSSV in water before it is being drawn into grow-out or hatchery facilities.

Several strategies for concentrating virus in water for detection including, the tangential flow filtration (TFF) with fetal bovine serum as stabilizing agent for the detection of infectious hematopoietic necrosis virus and infectious pancreatic necrosis virus by plaque assay [13], elution and skimmed milk flocculation procedure for the detection of human adenovirus, JC polyomavirus and noroviruses by qPCR [14]. In addition, glass fiber filter pre-coated with bovine serum albumin was used for the detection of marine birnavirus by PCR [15] and TFF for the detection of infectious salmon anemia virus by nested RT-PCR [16]. Centrifugation and membrane filtration, capacitive biosensor, combined ferric colloid adsorption and foam separation-based methods were employed to concentrate the WSSV in sea water for the detection by PCR [17-20]. In the recent study, TFF was used to concentrate the WSSV in shrimp pond water followed by PCR detection [21]. However, these methods are complex, sophisticated and costly for the field level detection of WSSV in shrimp culture. In addition, the differential results by PCR due to the variations in reaction conditions, specificity of primers, and presence of inhibitors [22, 23] made it unsuitable for the field detection. At present, there are no simple and easy methods available to detect WSSV in water, where the virus is present in very low concentration. In this study, we have developed a simple method of concentrating WSSV in water by alum flocculation, followed by rapid detection of virus using monoclonal antibody (MAb) based RapiDot.

## MATERIALS AND METHODS

### White spot syndrome virus preparation

Pacific shrimp, *Litopenaeus vannamei* showing signs of WSD were collected from shrimp farms of Nellore, Andhra Pradesh, India and brought to the laboratory on ice. WSSV infection in the diseased shrimps was confirmed by MAb (specificity to 28 kDa protein of WSSV) based immunodot [24]. The immunodot positive shrimps were used for the purification of WSSV [25] and re-confirmed by PCR [26]. Disease-free normal shrimps were collected from the farms of Kundapur, Karnataka, India for the preparation of negative control. A pair of pleopods and gills of each shrimp were fixed in absolute methanol and assayed for WSSV by two-step nested PCR [26].

### Experimental setup for concentrating WSSV in water by alum flocculation

To concentrate WSSV by alum through flocculation, freshly prepared semi-purified virus was diluted serially to 10^-10^ in TM buffer. One microliter from each dilution was subjected to I step and II step nested PCR [26]. The original stock of semi-purified virus (Stock) and the lowest dilution (Diluted) at which II step PCR detected the virus were tested for concentrating the virus by aluminium sulphate-Al_2_(SO_4_)_3_•16H_2_O (Nice Chemical Pvt. Ltd, India). A 50 ml capacity measuring cylinder containing 50 ml filtered (0.2 µm nitrocellulose membrane filter) seawater was added with alum at the concentrations of 15 and 30 ppm, and no alum addition served as a control. The diluted or original stock of semi-purified WSSV was added to the respective cylinders, mixed thoroughly and kept undisturbed to allow flocculation of WSSV by gravity. At every 2 h interval for the initial 12 h period and followed by 6 h interval till 36 h period, the floc deposited at the bottom in each treatment and control were collected completely using sterile sucker pipettes. The floc samples was centrifuged at 10,000 × g for 15 min at 4°C and the resultant pellet was resuspended in 100 µl PBS, labelled and stored at 4°C for further analysis. All the floc samples were analyzed by RapiDot [27], I and II step PCR [26].

### Experimental infection studies

*L. vannamei* free of WSSV were collected from farms around Kundapur, Karnataka, India and acclimatized to the laboratory condition for a week. Three shrimps (~14 g each) were stocked in aquarium tank containing 25 L seawater (salinity 27 ppt, pH 7.2, temperature 26°C) with continuous aeration. The acclimatized shrimps were injected with 100 µg semi-purified WSSV at the lateral position of third abdominal segment using 1 ml insulin syringe fitted with 30G × 5/16 (0.30 × 8 mm) needle. The injected shrimps were fed with commercial diet at 5% body weight and monitored daily for the development of clinical white spots on carapace and mortality. Semi-purified WSSV was prepared from the dead shrimps and added back to aquarium tanks, from which water samples were collected into 2 and 1 L beakers, and a 50 ml measuring cylinder for concentrating the virus by alum flocculation as per the protocol explained above. The white spot syndrome virus in the bottom floc samples was analyzed by RapiDot and II step PCR.

## RESULTS

### Confirmation of WSSV in purified samples

**Figure 1:**
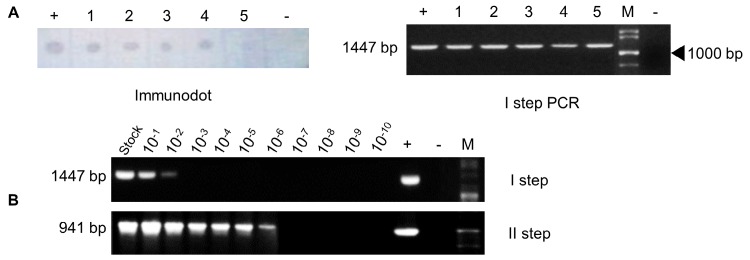
**Confirmation of WSSV in samples by Immunodot and Polymerase Chain Reaction**. **A**. Monoclonal antibody based immunodot assay and I step PCR were performed to confirm the presence of WSSV in infected *Litopenaeus vannamei* (1-5). The appearance of blue dots on the nitrocellulose membrane confirms the presence of WSSV in the tissue homogenate prepared from the gills and pleopods of infected shrimp. Similarly, the amplification of 1447 bp amplicon by I step PCR confirms the presence of WSSV in the semi-purified samples prepared from different *Litopenaeus vannamei* (1-5). **B**. Polymerase chain reaction confirms the presence of WSSV in serially log diluted semi-purified virus samples. The amplification of 1447 in 10^-2^ dilution and 941 bp in 10^-6^ dilution showed the presence of WSSV in serially diluted virus samples by I and II step PCR, respectively.

The semi-purified virus prepared from the immunodot-positive *L. vannamei* was further confirmed by I step PCR (**Fig. 1A**). The amplification of an amplicon size 1447 bp in I step PCR products that were electrophoresed on 1% agarose electrophoresis reaffirmed the presence of WSSV in semi-purified virus preparation. The preparations from normal shrimp showed no amplification of WSSV-specific amplicons by II step PCR.

### WSSV in water is concentrated by alum flocculation

**Figure 2:**
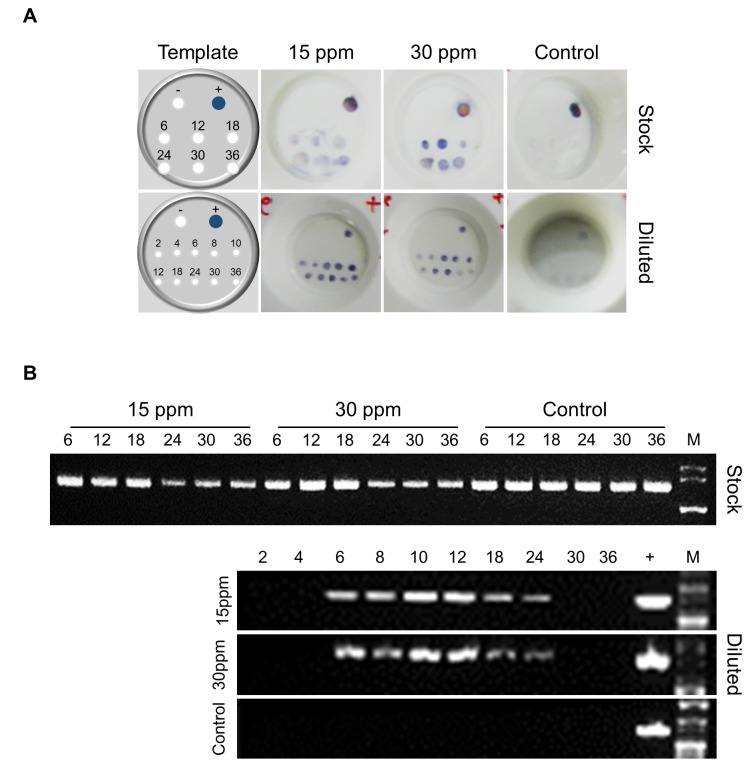
**Alum flocculation concentrates WSSV in water**. MAb based RapiDot (A) and I step PCR (B) analysis showing positive for WSSV in floc samples from stock and diluted virus flocculated by 15 and 30 ppm alum. M, molecular marker; +, positive Control; 6-36, hours of sampling.

Each log dilution of WSSV stock was analyzed by PCR and found the lowest dilutions detectable at 1 × 10^-2^ and 1 × 10^-6^ by I and II step PCR, respectively (**Fig. 1B**). The dilution of 1 × 10^-6^, in which II step PCR detected the virus was further used in the flocculation experiment and compared the results with original stock of virus flocculation by alum. The WSSV was detectable by RapiDot in floc samples from original stock of virus flocculated by 15 and 30 ppm alum compared to control. In RapiDot analysis, the intensity of dot gradually increased till 12 h flocculation of virus by 30 ppm alum and then decreased over time (**Fig. 2A**). However, I step PCR showed that the virus was detectable in all the floc samples, but the gel band intensity lowered after 18 h in alum floc samples compared to control (**Fig. 2B**). In addition, the WSSV was also detectable by RapiDot in floc samples from log-diluted virus flocculated by 15 and 30 ppm alum compared to control. The MAb based RapiDot could detect the WSSV very fast after 2 h of flocculation by alum and the dot intensity increased gradually until 24 h (**Fig. 2A**). However, I step PCR could detect the virus from 6 h of flocculation by alum and the gel band intensity lowered after 12 h in alum floc samples compared to control (**Fig. 2B**). Furthermore, the WSSV was not detectable by RapiDot in floc samples from both the controls added with diluted and original stock of virus, but without addition of alum. Moreover, I step PCR also could not detect the WSSV in floc samples of diluted virus control, with an exception, it detected the virus in floc samples from original stock virus control (**Fig. 2A** and **B**).

### Alum concentrates WSSV in water from experimentally infected shrimp Tanks

**Figure 3:**
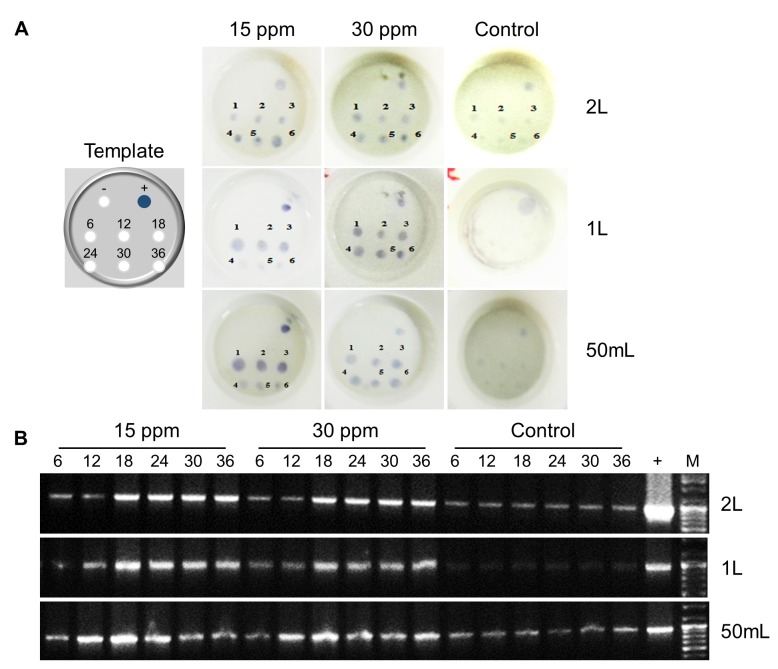
**Concentrating WSSV in experimentally infected water by alum flocculation**. MAb based RapiDot (A) and II step PCR (B) analysis showing positive for WSSV in floc samples from experimentally infected water in 2 and 1 L beakers, and 50 ml measuring cylinder added with 15 and 30 ppm alum to concentrate the virus. M, molecular marker; +, positive Control; 6-36, hours of sampling

Alum at 15 and 30 ppm flocculated the WSSV from experimentally infected shrimp water in different containers (2 L, 1 L beaker and 50 ml measuring cylinder). The WSSV was detectable by both RapiDot and II step PCR in all the floc samples at 6 to 36 h flocculation time with varied intensities (**Fig. 3A** and **B**). However, the dot intensity in RapiDot increased over the time for 2 L and 1 L beaker floc sample, but decreased with time for 50 ml measuring cylinder floc samples. Although II step PCR detected WSSV at all the time intervals but, the intense bands were observed at 18, 24, 30 and 36 h for 2 L, 18, 24, and 30 h for 1 L beaker samples, and 12, 18, and 24 h for 50 ml measuring cylinder samples. Further, RapiDot could not detect the WSSV in floc samples from control without alum addition to 1L beaker and 50 ml cylinder, with an exception, it detected the virus in floc sample from 2 L beaker with very faint dots. In similar, WSSV was not detectable in floc samples from 1L beaker by II step PCR, but very light bands showed that the virus was detected by II step PCR for floc samples from 2 L beaker and 50 ml cylinder. The WSSV detection by RapiDot and II step PCR in control samples could be due to the improper distribution of viral load from the dead shrimps or may attribute to the human errors during the sampling of water for analysis.

## DISCUSSION

Water is the major route of WSSV transmission into an aquaculture system [12]. WSSV is reported to be infective at least for 40 days at 30°C and 3-4 days in laboratory and pond conditions, respectively [28, 29]. The WSSV load of 1000 particles per ml of seawater was found to be viable and infective for up to 12 days**[30]. Therefore, the earliest and faster detection of WSSV in water is important and essential for the management with biosecurity measures to prevent its spread. In order to detect the virus from outbreak ponds, researchers have concentrated the virus from pond water and surrounding canal water by membrane filtration followed by PCR detection [18]. Although, the molecular methods are highly sensitive and specific, they are sophisticated, costly, complex and not suitable for routine field use. Hence, our laboratory and researchers from China and Thailand have developed simple, rapid and field-level diagnostic tools for the sensitive detection of WSSV in shrimp [27, 31, 32].

The virus detection in water has always been a challenge due to its low concentration. In this study, we tested the alum that is being routinely employed in drinking water treatment, for concentrating the WSSV in water and its detection by simple MAb based RapiDot. It is known that alum tend to flocculate the organic matter by enabling the aggregation of smaller organic/protein particles to floc that might be easier for the detection [33]. We made use of this principle of alum to test its ability to floc WSSV particles by employing 15 and 30 ppm concentrations to water inoculated with virus. The alum at both concentrations could concentrate the virus by flocculation as detected by RapiDot and PCR. The WSSV was detectable by RapiDot and I step PCR in floc samples from original stock of virus flocculated by 15 and 30 ppm alum. This might probably be attributed to the high concentration of semi-purified virus used for inoculation into water. In addition, RapiDot and I step PCR detected the WSSV in floc samples from log-diluted virus flocculated by alum. Interestingly, WSSV in floc samples from log-diluted virus sample, was detected four hour earlier by RapiDot than I step PCR. This result showed that MAb based RapiDot assay developed in our laboratory is simple easy and sensitive for the rapid detection of WSSV at lowest concentration through alum flocculation.

Further, this concept of concentrating virus by alum flocculation was evaluated by simulating the white spot disease outbreak in the laboratory aquaria through experimental infection of *L. vannamei* with WSSV injection. RapiDot and II step PCR detected the WSSV in floc samples within the first 6 h of alum addition at 15 and 30 ppm in 2, 1 L and 50 ml infected water. However, the intensity of dot increased over time of flocculation in all water samples with slight variations. The interesting point here is that we could floc the virus by alum in less volume of infected water, which is much lesser compared to the report of >60 L water for WSSV concentration and detection by PCR [21]. In addition to alum, several other agents have been tried by others to concentrate the virus, including TFF with skimmed-milk for adenovirus [14], glass-fiber filter pre-coated with bovine serum albumin for marine birnavirus [15], TFF for infectious salmon anemia virus [16] and TFF for WSSV [21]. However, these methods require laboratory, specific skills and equipment, which further makes the detection method costlier, time consuming and complex. Overall, a simple and easy method developed in this study for concentrating the WSSV by alum, as a flocculant and detection by a specific monoclonal antibody based RapiDot kit, would be useful for field-level detection of WSSV. Furthermore, aluminium sulphate can be effectively used to concentrate the WSSV through flocculation for simple detection at the farm-level.
